# The maturation of spinal dorsal horn nociceptive neuronal network activity over the postnatal period

**DOI:** 10.1097/PR9.0000000000001324

**Published:** 2025-09-03

**Authors:** Emma Battell, Neave Smith, Steve Woodhams, Laura Rich, Lucy Donaldson, Angus Brown, Charles Greenspon, Victoria Chapman, Gareth Hathway

**Affiliations:** a Physiology, Pharmacology and Neuroscience, School of Life Sciences, The University of Nottingham, Nottingham, United Kingdom; b Pain Centre Versus Arthritis, The University of Nottingham, Nottingham, United Kingdom; c Department of Neurology, School of Medicine, University of Washington, Seattle, WA, USA; d Department of Organismal Biology and Anatomy, University of Chicago, Chicago, IL, USA

**Keywords:** Pain, Spinal cord, Postnatal, Network, Multi-electrode array

## Abstract

**Introduction:**

The dorsal horn (DH) of the spinal cord is physiologically immature at birth. Spinal excitability increases and wide dynamic range (WDR) neurons in lamina V have lowered activation thresholds and larger receptive field sizes.

**Objective:**

The DH is composed of 5 laminae containing diverse interneuronal populations yet our understanding of the physiology of the DH is based on behavioural studies or extrapolation of single cell WDR recordings to the whole network.

**Methods:**

We have employed multi-electrode arrays to record contiguous activity across the whole DH in neonatal juvenile, adolescent, and young adult Sprague-Dawley rats after electrical stimulation of the ipsilateral hindpaw (0.01–5 mA).

**Results:**

Baseline DH activity increased significantly (*P* < 0.0001) over early life, as did DH responses in the post-stimulus period corresponding to A-fibre-mediated input. Age-dependent differences in DH activity during this period were not significantly different in the superficial and intermediate DH, but were in the deep DH. C-fibre-mediated activity was more variable and, unlike the A-fibre period, there were no significant differences in stimulus response profiles. However, significant C-fibre-evoked activity was detected in juvenile, adolescent, and adult groups across the whole array and particularly in the deep DH (*P* < 0.05 and *P* < 0.01). Finally, short-term plasticity (“wind-up”), previously observed with single-unit approaches was detected in all regions of the adult rat DH, but not in younger ages.

**Conclusion:**

Despite behavioural and single-cell responses being more excitable in early life, at a network level the DH is significantly less excited than in adulthood.

## Abstract 2

Supplemental Digital Content is Available in the Text.

Spinal neuronal network properties at rest, during innocuous and noxious stimulation, and following experimental synaptic plasticity are significantly altered during postnatal development.

## 1. Introduction

Pain impacts the lives of everyone regardless of age, sex, or ethnicity.^32^ The neurobiological mechanisms that detect, encode, process, and respond to painful stimuli are not hardwired at birth, undergoing profound maturation during early life.^14^

Pain arises due to activity in the peripheral nervous system, which is integrated into a series of interconnected pathways in the central nervous system. Pain in early life is characterised by lower somatosensory thresholds and exaggerated reflexes,^16^ which are, in part, due to immaturity in the spinal dorsal horn (DH).^4,21^

Sensory neurons have cell bodies in the dorsal root ganglia. Perinatally, these neurons lose spontaneous activity,^13^ with accompanying waves of changes in gene expression occurring over pre and postnatal development,^22^ including in sodium and potassium channel subunits and transient receptor potential channels.^51^

At birth, the laminar structure of the spinal cord is present and recognisable; however ,the DH continues to grow in the postnatal period.^29^ The adult DH is composed of 5 distinct laminae with differing primary afferent innervation. Central terminals of myelinated low-threshold fibres lie within a region extending from lamina I to lamina V with myelinated nociceptive terminals are found in laminae I–II, IV, and V. Unmyelinated terminals are found mainly in laminae I–II but C-low threshold mechanoreceptors are found throughout the DH.^1,9,25,47^ Each of these laminae contains diverse populations of interneurons and projection neurons, the latter conveying information to the brain.^19,42^

In vivo electrophysiological studies have significantly informed our understanding of maturing DH nociceptive processing. Single-unit recordings provide stable long-term data by focussing on wide dynamic range (WDR) neurons, which respond to all peripheral afferent fibre (PAF) types. However, these data fail to capture the broader population response and the diversity of spinal neuron phenotypes.

We use a new approach to study DH function, focusing on how the whole DH neuronal network changes over early life. Multi-electrode arrays (MEAs) allow simultaneous in vivo recording from all DH laminae, helping us understand the complexity of DH responses to noxious inputs without extrapolating between regions.^18^

## 2. Methods

### 2.1. Animals

Animal procedures were licenced by the UK Home Office (PB3DA999F) complying with Animals (Scientific Procedures) Act. Studies were approved by the University of Nottingham's Animal Welfare and Ethical Review Board. Male and female Sprague-Dawley rats between postnatal day (P) 9 and 60 were used age groups: neonatal (P9–11, n = 13); juvenile (P14–16, n = 16); adolescent (P21–23, n = 13); young adult (YA; P42–60, n = 13).

Time-mated females, sourced from Charles River UK, were housed with pups in individually ventilated cages at 21 ± 2°C and 55% humidity until weaning at P22. Postweaning, rats were housed 2 to 4/per cage. Animals were kept in a 12 h:12 h light–dark cycle, with food and water provided ad libitum.

### 2.2. Peripheral nerve recordings

Stimulus-evoked compound action potentials (CAPs) were recorded simultaneously from A and C fibres in sciatic nerves.^33^ Rats were killed by cervical dislocation, the sciatic nerve exposed and cut as it left the spinal cord. Both sciatic nerves were used and treated as separate experimental units.^34^ Dissected nerves were placed in a superfusion chamber maintained at 37°C using a TC-202 bipolar temperature controller (Digitmer, Welwyn Garden City, United Kingdom) and perfused with artificial cerebrospinal fluid (aCSF) bubbled with 95% O_2_ and 5% CO_2_ to maintain pH at 7.45.

The length of each sciatic nerve was measured and stimulated with a double pulse stimulator (Grass S88, A-M Systems, Washington). Compound action potentials were amplified 1000x, filtered at 30 kHz (Stanford Research Systems preamplifier, SR560, California), and acquired at 50 kHz using PClamp 10.7 (Molecular Devices software) via a Digidata 1440A analogue to digital converter (Molecular Devices). Data were analysed using Clampfit 10.7 (Molecular Devices, California) to determine CAP amplitude and normalised to the maximum response, to remove internerve variability. Latency to CAP peak was measured and divided by the nerve length to estimate conduction velocities.

### 2.3. Laminectomy

Spinal in vivo MEA electrophysiology was conducted as previously described,^18^ with some adaptations for working with pups. Animals were anesthetised (3% isoflurane, York, United Kingdom) until areflexic, transferred to a stereotaxic frame, and anaesthetic concentration reduced (2%). Body temperature was monitored and maintained at 37 ± 1°C via a homeothermic system (Harvard Apparatus, Massachusetts). A laminectomy and meniscectomy were performed, exposing the spinal segments L4/L5. Anaesthetic was then maintained at 1.6% for the duration of the experiment.

### 2.4. Electrode placement

A 16-channel linear MEA with 50-μm interelectrode spacing (A1 × 16-5 mm-50–177; NeuroNexus, Michigan) was positioned halfway along the anterior–posterior axis of the exposed cord (L4), over the lateral margin of the central vessel, using a micromanipulator (IVM Single; Scientifica, Clarksburg, NJ).

Stimulating electrodes, 30-gauge needles (Becton, Dickson, and Company, United Kingdom), were inserted under the skin of the hind paw, below the medial paw pads. An alignment protocol was run (3 mA, 50 ms, 0.1 Hz, x6) and evoked responses inspected for the characteristic local field potential (LFP) profile to confirm correct placement of the electrode. After making adjustments, alignment was checked again after 10 minutes.

#### 2.4.1. Age as a factor in dorsal horn size

The dorsal horn increases in size during postnatal development from ∼700 µm in P9 rats to 1000 µm in adult rats.^29^ We attempted to confirm the MEA location histologically by lesioning the spinal cord. Multiple lesioning protocols were attempted unsuccessfully. As an alternative, previous reports of dorsal horn laminae dimensions were reviewed (Table 1). From these studies, the electrodes expected to be in the superficial, intermediate, and deep dorsal horn regions were determined for each age group (Table 2).

**Table 1:** Dorsal horn laminae depths during postnatal development previously reported in the literature.

Age (d)	Depth of specified region (μm)								
	Whole dorsal horn	Whole dorsal horn (mean ± SEM)	LI	LIIo	LIIi	Sup. dorsal horn	Int. and deep dorsal horn	Sup. dorsal horn	Int. and deep dorsal horn
0	500	525 ± 7	43	39	65	147	380		
3								200	450/500
5		623 ± 4	45	42	68	155	470		
7	700								
10		692 ± 19	43	39	73	155	537	250	650/700
15	800	776 ± 17.5	41	44	84	169	525		
20		789 ± 13	42	42	90	174	620		
21								300	750/800
60	1000	1086 ± 5	42	47	93	182	905		
Ref.	^8^Measured spinal cord surface to ventral lamina VI	^21^Measured from surface of grey matter to central canal						^35^Measured from surface of spinal cord	

Depths from 3 previous studies are aligned to show depths of the whole dorsal horn and superficial (Lamina (L)I-IIi) and intermediate/deep (LIII–VI) dorsal horn depths between birth (day 0) and adulthood (day 60).

**Table 2:** The approximate depth (µm) of each electrode at all age groups.

Electrode	Neonate	Juvenile	Adolescent	YA
1			**50**	**50**
2	**50**	**50**	**100**	**100**
3	**100**	**100**	**150**	**150**
4	**150**	**150**	**200**	**200**
5	*200*	*200*	*250*	*250*
6	*250*	*250*	*300*	*300*
7	*300*	*300*	*350*	*350*
8	*350*	*350*	*400*	*400*
9	***400***	***400***	***450***	*450*
10	***450***	***450***	***500***	***500***
11	***500***	***500***	***550***	***550***
12	***550***	***550***	***600***	***600***
13	***600***	***600***	***650***	***650***
14	***650***	***650***	***700***	***700***
15	***700***	***700***	***750***	***750***
16		***750***	***800***	***800***
—				***850***
—				***900***
—				***950***

The value in the table represents the lower depth of the region in which the electrode is expected to record (eg, 50 represents 0–50 µm). Dorsal horn regions are (bold, italic, bold-italic) coordinated, bold representing the superficial laminae (LI–II), italic representing the intermediate laminae (LIII–IV), and bold-italic representing the deep laminae (LV–VI). Electrodes 1 and 16 are excluded during data analysis of P9–11 recordings, and electrode 1 is excluded from P14 to 16 recordings. Data taken from References 12, 29, 43.

P, postnatal day; YA, young adults.

### 2.5. Stimulation protocols

Dorsal horn neuronal activity in response to time-locked electrical stimulation of the hind paw was recorded. Stimuli of varying intensities (0.01–5 mA; 0.0167 Hz) were delivered via needle electrodes. Trains of 5 stimuli at each intensity were tested in ascending order and an intertrain interval of 10 minutes.^18^ To assess frequency-dependent increases in network excitability, a wind-up protocol was also used in the same animals. The low-frequency control train (20 × 5 mA, 2 ms, 0.1 Hz) was followed by 3 wind-up trains at higher frequency (5 mA, 2 ms, 0.5 Hz) with 15-minute intertrain intervals.

### 2.6. Data extraction and analysis

The array was connected via a custom flexible connector (Omnetics, Minneapolis, MN) to a headstage (HSt/16o25‐18p‐xR; 1× gain; Plexon, Dallas, TX). This was connected to a Private Branch Exchange preamplifier (1000x gain; Plexon), which split and filtered the raw data by frequency into LFP (0.7–300 Hz, 5-kHz sampling frequency) and high-frequency data (150 Hz–8 kHz, 40 kHz). Local field potential data were sent directly to a computer, whereas high-frequency data were sent via a Multichannel Acquisition Processor (Plexon). The Plexon Sort Client program was used to set sorting parameters and visualise waveform activity at each electrode (Supplementary Fig. 1, http://links.lww.com/PR9/A337). Local field potential data were continuously sampled, but high-frequency waveforms were captured when a depolarisation of ≥10% from baseline was detected. The depolarisation threshold and refractory period (1200 μs) were as used previously^18^ and recommended by Plexon. Local field potential data were used exclusively to confirm correct alignment of the array.

Data were exported to MATLAB 2022 (The MathWorks, Natick, MA) for processing. Supplementary Figure 1, http://links.lww.com/PR9/A337 illustrates unsorted multi-unit data acquired at each channel on the MEA. The large variability in responses at different contacts can be clearly seen by comparing the highlighted channel (A) to the other 15-channels (B). Depolarisations ≥10% below baseline at each electrode were captured to produce raster plots of threshold crossings (C).

Custom MATLAB scripts were used to isolate peri-stimulus data, sort threshold crossings into appropriate epochs for each age group, and analyse spatial and temporal patterns of activity.

Regional activity was determined by meaning threshold crossings across electrodes recording within each region of the DH during each epoch. As the DH changes in size over the postnatal period, different numbers of electrodes were used to record each of the 3 regions in different age groups, and meaning responses across electrodes was, therefore, the logical way of ensuring that age-based comparisons could be made.

Data analysis was performed using Prism 10 (GraphPad Software, La Jolla, CA). Statistical comparisons were made using the mean response of 5 applications of each stimulus to each animal. Group sizes were determined based on previous data collected in adult SD rats (Greenspon et al., 2019). Unless otherwise stated, all statistical tests were parametric. Regional and stimulation responses at individual ages were compared via 2-way repeated measure (RM) ANOVAs followed by Tukey post-hoc tests.

## 3. Results

### 3.1. Sensory neuron electrophysiological profiles vary significantly across postnatal development

**Figure 1.**
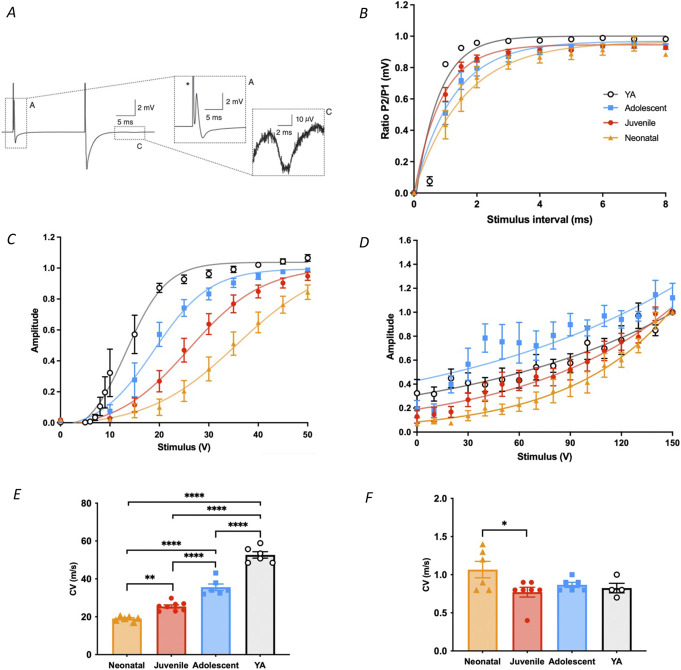
The physiological properties of A and C fibres across postnatal development. (A) Example of a compound action potential from rat sciatic nerve. (B) Compound action potentials (CAP) evoked by a double pulse protocol with the second pulse (P2) occurring at decreased interpulse intervals from the first (P1). The ratio of P2/P1 is plotted for the second peak against the interpulse interval. Through postnatal maturation, a smaller interpulse interval is needed to decrease the P2 peak (B). Stimulus response plots for A and C fibres (C and D). Conduction velocity (CV) of A fibres (E), but not C fibres (F), increases with postnatal age. CV is shown in m/s values. Each point represents recordings from 1 sciatic nerve, bars represent the mean ± SEM for each age group. Asterisks represent significant responses between ages after a 1-way ANOVA and post-hoc Tukey test.

A- and C-fibre properties change with age. Compound action potentials were measured by electrically stimulating sciatic nerves using a double-pulse protocol. The first pulse (15V) recruits A fibres, and the second pulse (130V) recruits C fibres (Fig. 1A). Compound action potential profiles for A and C fibres were distinguishable in all ages. To measure voltage-gated sodium channel inactivation, 2 supramaximal pulses were applied with decreasing interpulse intervals. Neonates required a longer interval for full Na+ channel recovery compared to young adults (Fig. 1B).

Stimulus response profiles for CAP amplitude in the A-fibre epoch (Fig. 1C) showed a leftward shift with age. There is full A-fibre recruitment in the oldest group, YA, above 20V. Our data show the same general trend for the C-fibre stimulus-response profiles, but this is not as robust due to the thousand-fold decrease in C-fibre CAP amplitude compared to the A-fibre CAP (Fig. 1D).

There was a significant increase in A-fibre conduction velocity (CV) as animals aged (F_4,27_ = 101.2; *P* < 0.001 ANOVA Tukey post-hoc test; Fig. 1E). Neonatal A-fibre CVs were significantly slower than all other age groups (*P* < 0.01 vs juvenile, *P* < 0.0001 for all other comparisons). Age significantly effected C-fibre CV (F_4,24_ = 3.312 *P* = 0.0270 ANOVA). Conduction velocities measured in this epoch were significantly faster in neonates vs juvenile rats (*P* = 0.0251 Tukey post-hoc test) but not when compared to the other ages (Fig. 1F).

These CVs were used to determine the expected A- and C-fibre latency periods at each postnatal age for subsequent MEA studies (Table 3). There is a consistent calculated A-fibre onset, varying <1 milliseconds between age groups, but increasingly delayed C-fibre latency periods across postnatal development.

**Table 3:** Peripheral afferent fibre latency periods throughout the life course.

Age	Hind paw to recording site distance (cm)	Latency onset calculated from CV (ms)		Latency range used (ms)	
		A fibre	C fibre	A fibre	C fibre
Neonatal	5.5	2.9	51	3–50	50–250
Juvenile	6.6	2.6	77	3–50	75–275
Adolescent	8.8	2.4	97	3–50	95–295
YA	11	2.1	145	3–50	120–320

The approximate distance between the hind paw where stimulus application occurred to the spinal recordings site increases with age as expected. The calculated onset for the A- and C-fibre latency periods during the activity evoked by each of these fibre types in the DH and the range of latency periods used for subsequent analysis are shown in the rest of the table.

CV, conduction velocity; YA, young adult.

### 3.2. Region-specific changes of A-fibre inputs in the maturing dorsal horn

**Figure 2.**
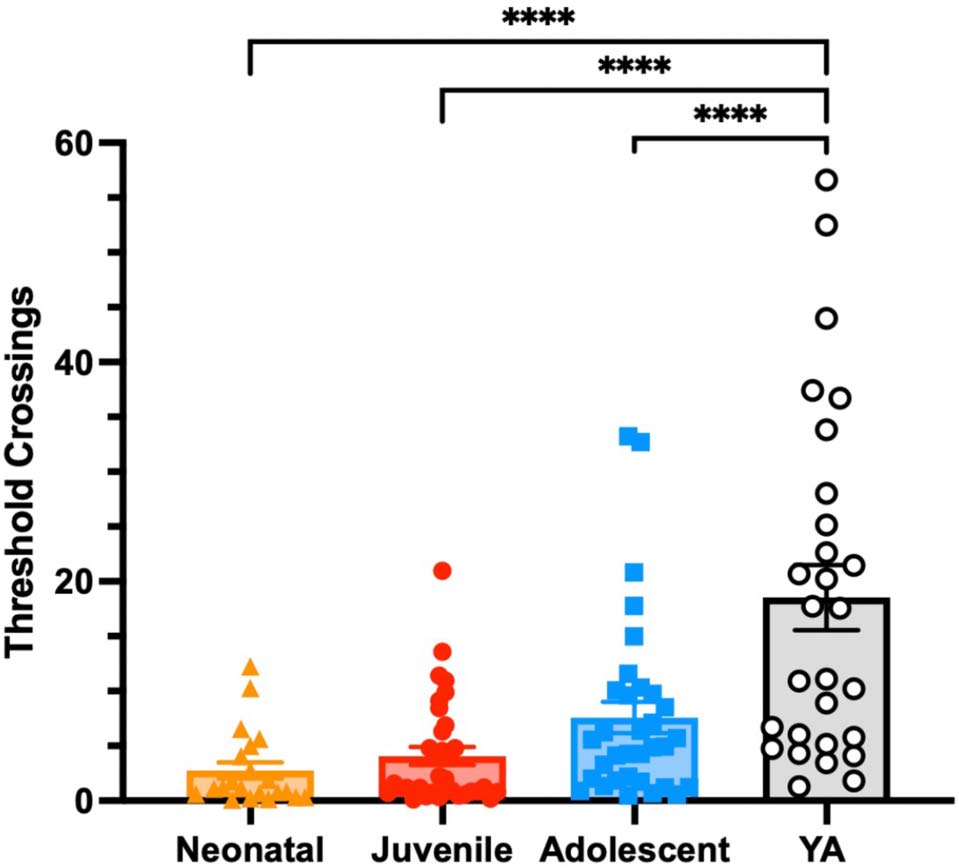
Baseline DH activity significantly increases with postnatal age. Threshold crossings over 60 seconds before the first stimulation delivered using mean activity recorded across the whole array. Individual data points represent an individual rat with bars representing mean ± SEM. Age had a significant effect (*P* < 0.0001) on the baseline activity recorded between the neonatal (n = 21), juvenile (n = 34), adolescent (n = 33), and young adult (YA) (n = 28) rats. DH, dorsal horn.

We next investigated differences in nonstimulated neuronal activity between age groups across the whole array (WA) during the prestimulus period, to see if on-going unstimulated activity differed between age groups. Spontaneous threshold crossings were significantly higher in YA compared to neonates, juveniles, and adolescents (Fig. 2) (F_3,12_ = 15.94; *P* < 0.0001, ANOVA with Tukey post-hoc test). No significant differences were found between the younger groups.

**Figure 3.**
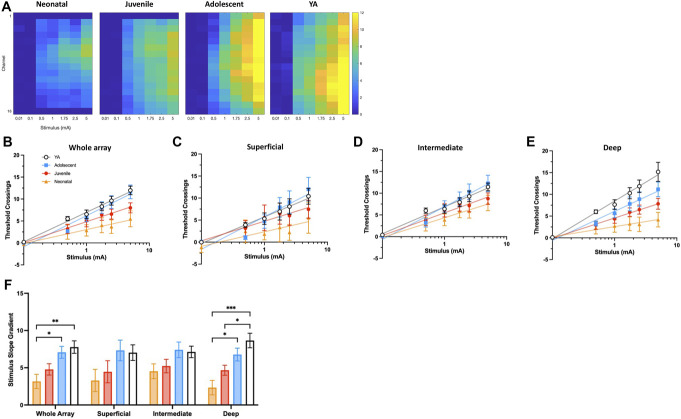
Age-dependent differences in A-fibre response occur in the DH after electrical stimulation of the hind paw. (A) Heatmaps of summed activity in the A-fibre latency period (3–50 ms poststimulation) at each electrode (*y*-axis) at 0.1, 0.5, 1, 1.75, 2.5, and 5 mA (*x*-axis). Colours represent mean number of threshold crossings (n = 4–6). Electrodes are in order: 1 is the most dorsal and 16 the most ventral. For neonatal rats, electrodes 1 and 16 are removed and electrode 1 is removed in the juvenile rats, to better represent the size of the dorsal horn at these ages. Increasing intensity of evoked activity is depicted as colour moves from deep blue, through green, into more intense yellow. Electrodes in the superficial DH are shown at the top of each plot, and stimulation intensity is on the *x*-axis. These data reveal marked differences in spatial responses of the DH at different stages of postnatal development. Responses in neonatal rats are smaller than those seen in YA rats. In neonates, evoked activity was restricted in dorso-ventral span (which can be visualised on the *y*-axis) compared to the older age groups, with notably lower activity in the superficial and deep DH. Summed DH activity (number of threshold crossings) is shown in the A-fibre epoch across the whole array (B), superficial (C), intermediate (D), and deep (E) regions. The stimulus gradient (F), obtained from (B–E), respectively, was used as a measure of stimulus encoding at the different ages. Bars and data points represent mean ± SEM *P < 0.05, **P < 0.01, ***P < 0.001 after 2-way repeated measures ANOVA with Tukey post-hoc test. DH, dorsal horn; YA, young adult.

Age-dependent differences in A-fibre response after hind paw stimulation were observed across the DH. Neonatal rats showed smaller and more restricted evoked activity compared to YA rats (Fig. 3A).

Next, we examined stimulus-encoding changes in DH networks during the A-fibre epoch to understand physiological changes across early life. Significant age-related differences in DH processing were found across the WA (Fig. 3B) (F_3,17_ = 6.160, *P* = 0.005), with neonatal responses localised superficially compared to those of adolescents (*P* = 0.0236) and YA (*P* = 0.0071). In the superficial (Fig. 3C) and intermediate (Fig. 3D) DH, there were no significant age-related differences. In the deep DH, significant effects of age were observed (Fig. 3E) (F_3,17_ = 9.572, *P* = 0.0006), with neonates differing from adolescents (*P* = 0.0118) and YA (*P* = 0.0005), and juveniles differing from YA (*P* = 0.0187). Statistical comparisons of slope gradients are shown in Figure 3F.

Using this low-frequency stimulation approach (1 electrical stimulation per minute, 0.0167 Hz), we assessed whether there was any sensitisation of network activity in the A-fibre epoch. We found no evidence for this in any age group in response to any stimulation intensity (Supplementary Fig. 2A-D, http://links.lww.com/PR9/A337).

### 3.3. Dorsal horn changes are less prominent during C-fibre activation

**Figure 4.**
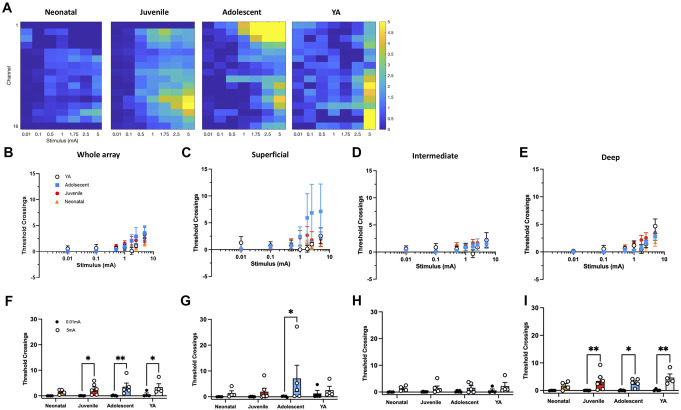
Electrical C-fibre stimulation produces difference response profiles through postnatal maturation. Heatmaps of mean activity in the C-fibre latency period (200 ms, the onset dependent on age) at each electrode (*y*-axis) at 0.1, 0.5, 1, 1.75, 2.5, and 5 mA (*x*-axis). Colours represent mean number of threshold crossings (n = 4–6), relative to the colour bar on the right. Electrodes are in order: 1 is the most dorsal and 16 the most ventral; for neonatal rats, electrodes 1 and 16 are removed and electrode 1 is removed in the juvenile rats, to better represent the size of the dorsal horn at these ages. Summed DH activity (number of threshold crossings) in the C-fibre epoch across the whole array (B), superficial (C), intermediate (D), and deep (E) regions. C-fibre response to 0.01 mA vs 5 mA across the whole array (F), superficial (G) intermediate (H), and deep (I) DH. Bars and data points represent mean ± SEM *P < 0.05, **P < 0.01, after 2-way repeated measures ANOVA with Tukey post-hoc test. DH, dorsal horn.

We examined electrically evoked activity in the C-fibre epoch. Heatmaps suggested regional differences in DH C-fibre latency responses among younger age groups compared to YA (Fig. 4A). However, quantifying these responses showed less clear age differences and nonlinear encoding of C-fibre activity (Figs. 4B–E).

To assess significant C-fibre–mediated evoked activity, we compared responses to subthreshold 0.01 mA and suprathreshold 5-mA stimuli across age groups. Significant C-fibre–evoked activity (0.01 vs 5 mA) was detected in all age groups except neonates (Fig. 4F). In the superficial DH, only adolescents showed a significant increase in C-fibre–evoked activity (Fig. 4G). No significant activity was found in the intermediate DH (Fig. 4H). In the deep DH, significant increases were observed in all age groups except neonates (Fig. 4I).

### 3.4. Age-dependent effects on wind-up in the dorsal horn

**Figure 5.**
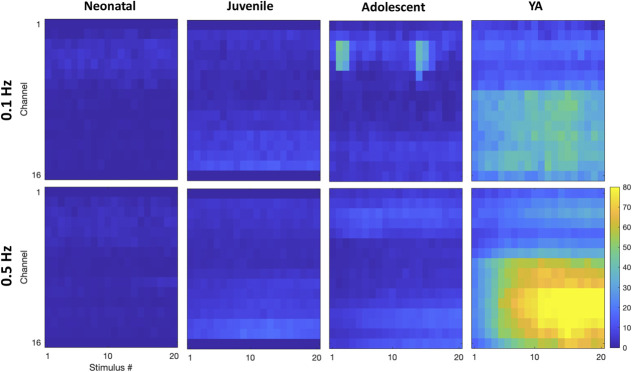
Age-dependent changes occur in the DH response after wind-up stimulation of the hind paw. Heatmaps of C + postdischarge (C + PD) latency activity after each stimulus (A). Control train is 20 stimuli at 0.1 Hz and the wind-up train is 20 stimuli at 0.5 Hz (both of 5 mA stimuli for 2 ms). Each cell represents summed latency activity at each channel for the stimulus. Colours represent mean number of threshold crossings (n = 5–6), relative to the colour bar on the right. Electrodes are in order: 1 is the most dorsal and 16 the most ventral. For neonatal rats, electrodes 1 and 16 are removed, and electrode 1 is removed in the juvenile rats, to better represent the size of the dorsal horn at these ages. DH, dorsal horn.

**Figure 6.**
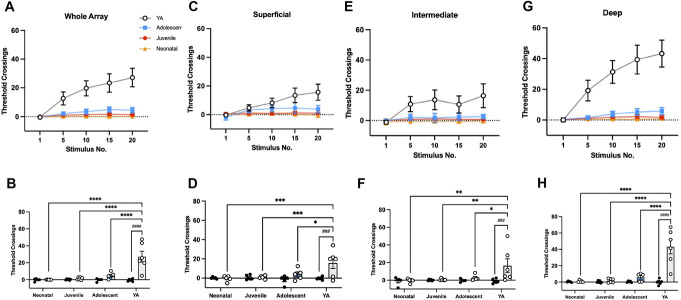
Age-dependent changes occur in the DH response after wind-up stimulation of the hind paw during the C + PD latencies. Wind-up of DH neurons, a model of synaptic plasticity, is significantly different between postnatal rats and YA. Wind-up was observed across the whole array (A) as well as in the superficial (C), intermediate (E), and deep (G) laminae during the C-fibre and postdischarge latencies after 0.5-Hz stimulation in YA but not in the younger ages. Stimulus 1 vs 20 at each age group across the whole array (B), superficial (D), intermediate (F), and deep (H). Bars and data points represent mean ± SEM, *P < 0.05, ***P < 0.001, ****P < 0.0001 (between age comparison), and ###*P* < 0.001 control vs wind-up after 2-way repeated measures ANOVA with Tukey post-hoc test. DH, dorsal horn; YA, young adult.

Plasticity in spinal networks is an important physiological property, which underlies central sensitisation. We investigated short-term changes in synaptic efficacy in the maturing DH using a wind-up protocol. Control trains of electrical stimuli (20 × 5 mA, 2 ms, 0.1 Hz) applied to the plantar foot pad did not induce wind-up at any age (Fig. 5). Increasing the frequency to 0.5 Hz induced significant wind-up in the deep DH of YA, but not in younger ages (Fig. 5). A 2-way repeated measures ANOVA showed significant effects of age (F_4,71_ = 13.21, *P* < 0.0001) and stimulus (F_3,18_ = 10.57, *P* = 0.0003), with a significant interaction (F_12,71_ = 8.070, *P* < 0.0001) on WA responses during the C-fibre + postdischarge epochs (C + PD, Fig. 6A). Responses increased significantly between stimulus 1 and 20 in the YA group (Fig. 6B), but not in younger ages. Young adult rats exhibited significantly greater wind-up compared to all other ages (Fig. 6B; *P* < 0.001).

Regional analyses showed a similar pattern in the superficial DH (Fig. 6C), with significant effects of age (F_4,71_ = 6.608, *P* < 0.0001) and stimulus (F_3,18_ = 3.695, *P* = 0.0312), and a significant interaction (F_12,71_ = 3.695, *P* < 0.0003). Significant wind-up occurred only in the YA group (Fig. 6D; *P* < 0.05 to *P* < 0.0001), with greater responses to stimulus 20 when compared to younger ages.

In the intermediate DH (Fig. 6E), there was a main effect of stimulus (F_4,71_ = 4.611, *P* = 0.0023) but not of age. A *post-hoc* Sidak multiple-comparison test showed significant increases from stimulus 1 to 20 in the YA group (Fig. 6F; *P* < 0.001), with differences at stimulus 20 between YA and younger groups (*P* < 0.05 YA vs adolescent, *P* < 0.01 YA vs neonate or juvenile).

In the deep DH (Fig. 6G), main effects of age (F_3,18_ = 14.55, *P* < 0.0001) and stimulus (F_4,72_ = 14.91, *P* < 0.0001) were observed, with a significant interaction (F_12,72_ = 10.23, *P* < 0.0001). Young adult rats showed significant increases in network activity between stimulus 1 and 20 (Fig. 6H) and compared to younger ages (*P* < 0.0001).

**Figure 7.**
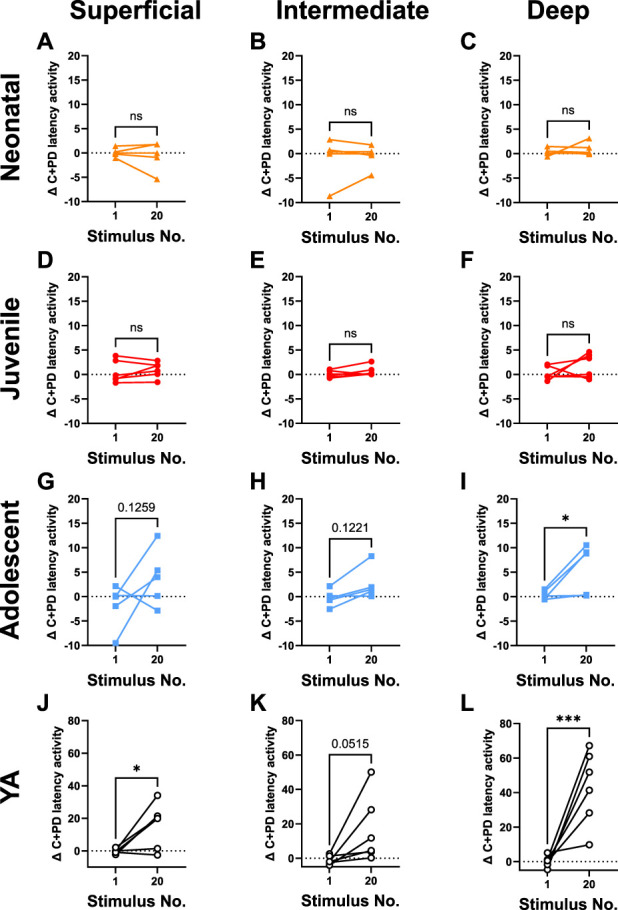
Regional analysis of age-dependent changes in DH wind-up. Regional analysis reveal no significant increase in activity in the C-fibre and postdischarge latencies in neonatal (A–C) or juvenile (D–F) rats across the DH after wind-up stimulation of the hind paw (20 × 5 mA, 2 ms, 0.5 Hz). In contrast, in adolescent rats (G–I), a significant increase in activity was observed in the deep DH (I), with a trend towards wind-up in the superficial (G) and intermediate (H) DH. In young adult rats (J–L), activity increased in all 3 regions, with significant wind-up in the superficial region (J), but most prominently in the deep (L) DH. Note the much larger increase in activity in YA compared to the younger age groups. Data represent the change in threshold crossings compared to the corresponding stimulus in the control train (20 × 5 mA, 0.1 Hz) in the C-fibre & postdischarge latency periods for each age group (Table 1). DH, dorsal horn; YA, young adult.

A 2-way ANOVA found no significant wind-up in neonatal, juvenile, or adolescent groups, although there was a trend towards increased C + PD latency responses in adolescents. Separate comparisons of stimulus 1 and 20 within (but not between) each age group showed no significant wind-up in neonatal (Figs. 7A–C) or juvenile (Figs. 7D–F) rats. Some adolescents showed wind-up in the superficial (Fig. 7G) and intermediate (Fig. 7H) DH, but only the deep DH responses were significant (Fig. 7I). In YA, significant wind-up was observed in the superficial (Fig. 7J; *P* < 0.05) but not in the intermediate layers (Fig. 7K). Significant wind-up was seen in the deep DH (Fig. 7L; *P* < 0.05). No wind-up of A-fibre responses was observed (Supplementary Fig. 3, http://links.lww.com/PR9/A337).

## 4. Discussion

We show considerable differences in DH network responses to somatosensory inputs during postnatal development in the rat. The overall environment of the immature DH appears to be one of limited activity, with neuronal responses across the DH being significantly lower at rest and after electrical stimulation of the hind paw.

### 4.1. The changing physiological properties of peripheral afferent fibres over early life

A- and C-fibre waveforms were detected at all ages; this was unsurprising as these can be detected from birth, although in an immature form.^12^ The A-fibre conduction velocities and amplitudes increased with postnatal age between neonates and young adults, supporting previously reported findings.^28^ C-fibre conduction velocity was most varied in the youngest P9–11 age group but appears faster than at later stages of development, in line with previous observations that C-fibres are immature in neonatal and adolescent rats.^15^

Polymodal nociceptors are found before birth and can be distinguished from low-threshold fibres.^13^ The stimulus-response characteristics of low- and high-threshold fibres mature over postnatal life with C-fibre properties modulated by exposure to NGF.^13,26^ Subtypes of A fibres decrease in number over the first 10 days of postnatal life.^50^ These changes in physiological properties result from developmental alterations in expression of ion channels, for example NaV1.8 (and others), which occur over the first 1 to 2 weeks from birth.^7^

### 4.2. Resting network activity increases over early life

Our data clearly demonstrate a significant increase in basal DH activity across postnatal maturation. There is a lack of in vivo data regarding background neuronal activity in the maturing DH. Traditionally, resting firing properties of single units are not reported in the literature. Some ex vivo studies of superficial DH neurons^3^ in isolated spinal preparations observed increasing spontaneous activity across the first 10 postnatal days. Our data support this, showing that this extends beyond the first 2 weeks of life.

Although age is the most obvious factor differentiating the experimental groups in our study, state-dependence (resting activity) can be dependent on other factors. We have used a widely adopted anaesthetic protocol, the same (minus the inclusion of N_2_O) as that used in single-unit DH recordings.^17,36^ Extracellular recordings are impacted by volatile anaesthetics, with nociceptive specific neurons more susceptible than WDRs.^5^ By recording from all populations simultaneously, our unbiased MEA approach assesses integrated network responses to account for this heterogeneity in response.

Age significantly affects the sensitivity of acute neuronal responses to anaesthetics. Chang found that isoflurane impacts spontaneous cortical activity at P7 but not at P14 and older, although responses to noxious stimuli were consistent across ages.^10^ The minimum alveolar concentration (to assess equipotency of agents across ages) needed to prevent limb movement to noxious stimuli is higher in P7 rats than older ages, indicating lower sensitivity in neonates and that spinal and supraspinal sensitivities differ. This suggests a complex pharmacology of volatile anaesthetics on spontaneous vs evoked network activity. It should be noted that many previous studies^15,16,35,37–39,43^ have also used a very similar approach to anaesthesia to that employed here.

### 4.3. Dorsal horn network activity during the A-fibre epoch

Our data show age-related differences in DH network activity during the A-fibre epoch. Activity was evoked in all age groups, with increased dynamic range of evoked threshold crossings as age increased. Stimulus intensity encoding was present in all ages and regions, with clear differences most evident in the deep DH.

Previous studies show that latencies to A-fibre stimulation decrease with age but did not report the magnitude/duration of responses.^24^ Receptive field size of DH neurons decreases with age,^43^ indicating changes in the balance of synaptic inhibition and excitation.

Thresholds to evoke cutaneous withdrawal reflexes are significantly lower in neonates, and the magnitude and duration of reflex withdrawals also decrease.^2,20,45^ This suggests greater excitability in the DH network response, yet our data do not support this. Our MEA approach samples neuronal populations randomly, not targeting specific neuron types^4^ and does not extend to the ventral horn and, therefore, does not record motoneurons. Cutaneous withdrawal reflexes are polysynaptic with sensory input and activation of spinal motoneurons linked by one or more interneurons.^27^ Enhanced behavioural responses in early life concomitant with reduced MEA activity in the DH might be explained by immature inhibitory DH signalling and/or integration of interneuronal populations into DH networks. Although immature inhibitory neurotransmission in the DH is known,^4,8,16,20,43^ our random sampling of DH neurons prevents us from attributing our data to this.

A-fibre termination patterns in the superficial DH refine in an activity-dependent manner across early life.^6,49^ Xu et al. found that disrupting microglial synapse engulfment during this period affects responses to dynamic mechanical stimuli. We only studied responses to electrical stimulation, finding no evidence of A-fibre sensitisation at different intensities or frequencies. Further research is needed to explore the interaction between DH responses and A-fibre synapse refinement during development.

### 4.4. Dorsal horn network activity during the C-fibre epoch

Significant C-fibre–evoked activity was detectable across all regions of the DH except the intermediate laminae at all ages. There is considerable evidence indicating a lack of C-fibre–evoked activity in the neonatal DH,^12,13,15^ although C-fibre terminals are present in the DH during the embryonic period.^23^ The lack of evoked network activity during the C-fibre epoch illustrates that functional C-fibre inputs in neonatal rats are insufficient to provoke postsynaptic responses.

We calculated the latencies for A- and C-fibre activity in the DH based on sciatic nerve conduction velocities. A-fibre latency, typically divided into Aβ- and Aδ-fibre periods, was grouped together resulting in a consistent onset time varying by only 1 milliseconds across ages. This approach has been used previously.^44^ C-fibre latency periods increased with age, likely due to the growing distance between the hind paw and recording site. These latencies agree with previous studies suggesting specific periods for A-fibre (0–50 ms) and C-fibre (50–250 ms) recordings in pups.^44^ A-fibre latency responses were consistently between 2 and 3 milliseconds, decreasing slightly with age due to myelination, leading to a chosen period of 3 to 50 milliseconds for all ages. C-fibre latency reflected MEA recordings at all postnatal ages, with young adult animals showing a slightly later onset than previously reported, allowing a set epoch period of 200 milliseconds for all ages.^44^

### 4.5. Plasticity in maturing dorsal horn networks

Synaptic plasticity in the DH underpins changes associated with chronification of pain.^48^ A widely used experimental model of synaptic plasticity, known as wind-up, has been used extensively in the literature to investigate WDRs.^30,41^ We demonstrate no significant network wind-up in the maturing DH, either across the whole array or regionally, until the YA age group. Previously, it has been shown that wind-up could be evoked in 18% of WDR cells at P10, increasing to 40% of cells by P21.^16^ These data are not in total accordance with our own, but it is important to note that we are measuring “wind-up” of a whole network of neurons, whereas all previous studies have only assessed individual neurons. Only WDR neurons are capable of wind-up, with nociceptive specific neurons not demonstrating this form of synaptic plasticity.^11^ The lack of network-wide wind-up in younger age groups does not imply that WDR neurons in these ages do not undergo potentiation.

It is also clear from other studies that nociceptive responses in younger rats can be evoked by either application of inflammogen^45^ or surgical injury,^46^ and that this produces a persistent nociceptive state. It may be that, due to the short period in which wind-up stimuli are applied compared to the longer periods of pain evoked by capsaicin,^45^ carrageenan,^43^ or surgical incision,^46^ a different pattern of network activity arises.

Our experimental approach offers a new way to study DH physiology by focusing on population/network responses rather than individual cells. Although it provides a snapshot of DH activity, it has limitations, such as the inability to record from multiple identifiable single neurons over long periods, and the inability to differentiate between inputs from cutaneous and muscle afferents. Future research aims to classify neurons into subpopulations and modify their activity using opto- or chemogenetic approaches as well as employing higher density probes (such as is achieved in the brain)^40^ both of which will permit identification of evoked activity from specific cutaneous afferent fibres over longer periods of time. Currently, we use a single-shank MEA to sample DH networks in one plane, but evoked activity also varies in other planes.^31^ This work highlights the complex organization of DH network responses, influenced by the somatotopic organization of DH neuronal networks and PAFs, which may change with age or in acute or chronic pain states.

In conclusion, we show age- and region-specific differences in the processing of afferent inputs from distinct sensory neuron inputs to the maturing dorsal horn. We demonstrate substantial differences in whole DH neural network activity, rather than relying on individual neuronal responses. We show that network plasticity is also differentially regulated across postnatal development. Multi-electrode arrays represent a new tool to further elucidate how the DH processes and responds to pain, and an exciting technology to interrogate fundamental neurophysiology and the way drugs and therapeutics alter network activity in the DH.

## Disclosures

The authors have no conflict of interest to declare.

## Supplemental digital content

Supplemental digital content associated with this article can be found online at http://links.lww.com/PR9/A337.

## Supplementary Material
