# Supplementary material for: The maturation of spinal dorsal horn nociceptive neuronal network activity over the postnatal period
Source: Pain Rep. 2025 Sep 3;10(5):e1324. doi: 10.1097/PR9.0000000000001324 (PMC12410310; doi:10.1097/PR9.0000000000001324)
Supplement: Supplementary file 1 [file painreports-10-e1324-s001.pdf]

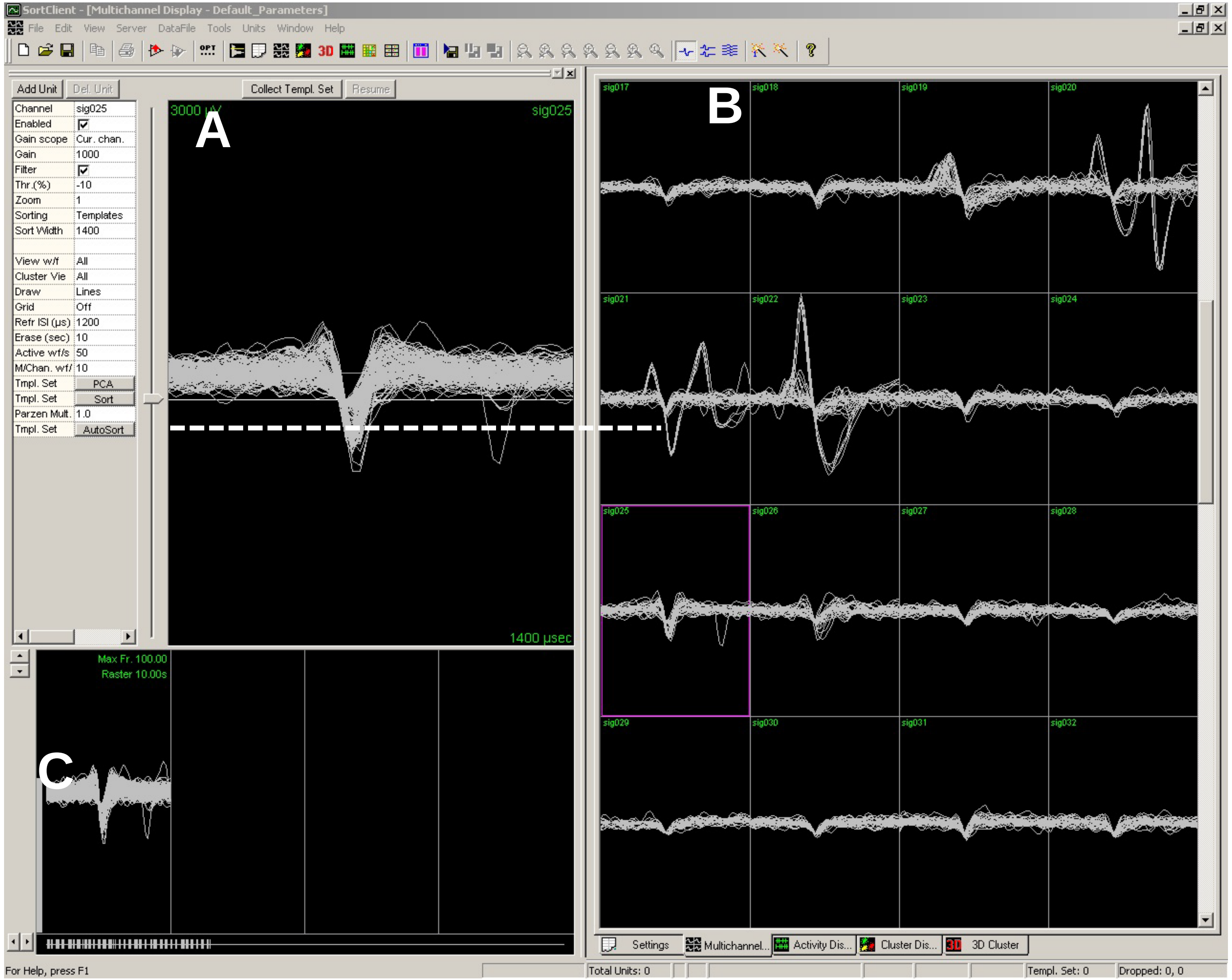

Supplementary Figure 1:
Screenshot of data acquisition. **A** Depicts multi unit activity detected at a single electrode from the centre of the array, with the 10% threshold limit shown by horizontal dotted white line. **B** Activity at all 16 contacts on the multi-electrode array. Note the large variability in activity levels and size of responses between electrodes. The single contact depicted in **A** is highlighted by a purple box. **C&D** Data acquisition, threshold crossings are detected from each unit (**C**) and displayed as a raster plot (**D**). Time signatures of threshold crossings are recorded.

Lack of sensitization of whole array A fibre latency responses with repeated stimulation

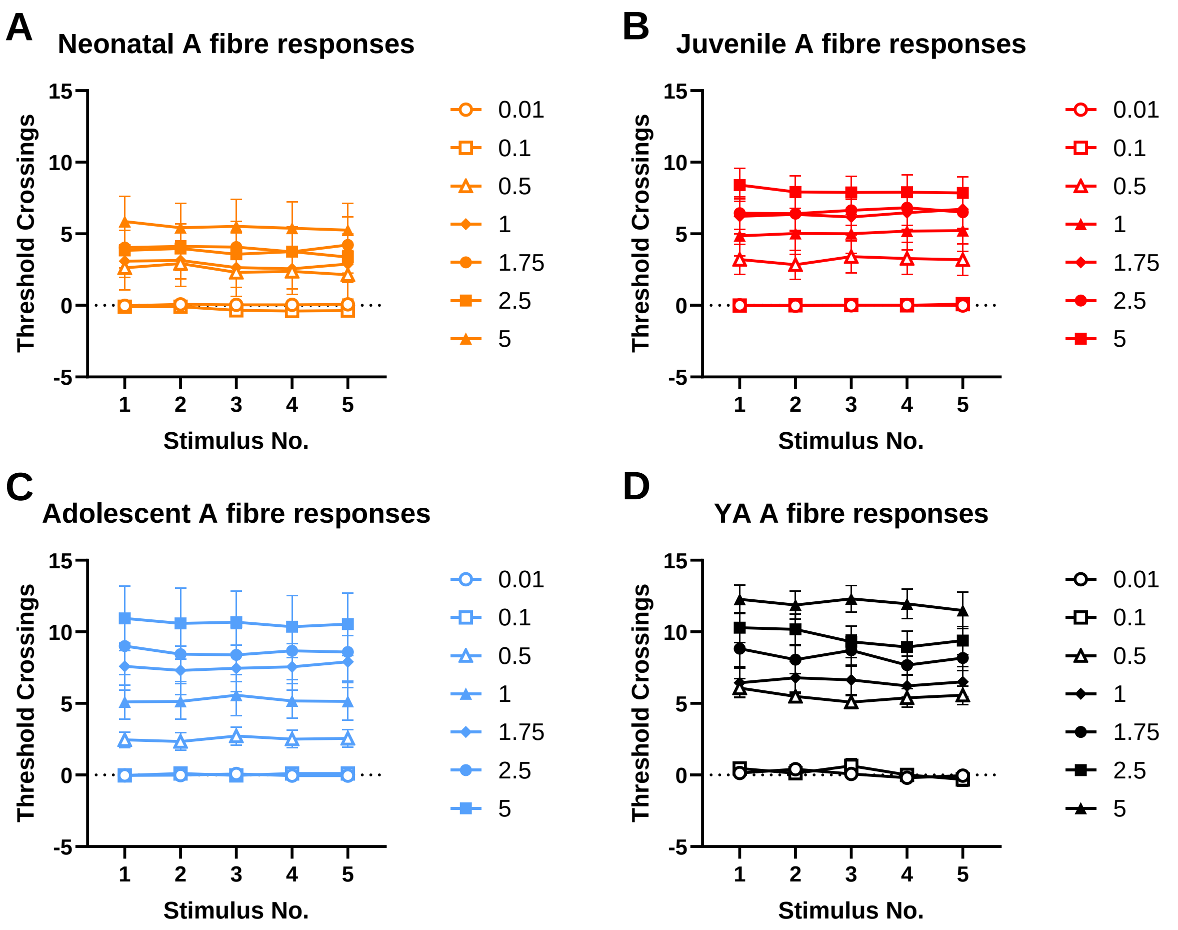

Supplementary Figure 2: Lack of sensitization of A fibre latency responses following repeated low frequency stimulation of the hindpaw in all age groups. Responses did not vary significantly within trains of repeated stimulation (0.01-5mA, 5x 2ms, 0.016Hz) at any of the stimulus amplitudes assessed in neonatal (**A**), juvenile (**B**), adolescent (**C**), or young adult (**D**) animals. Data represent summed threshold crossings in the A latency period averaged across the whole array.

Add 1mA 1st vs 5th for all age groups from electrical data

Lack of sensitization of whole array A fibre latency responses to repeated high frequency stimulation

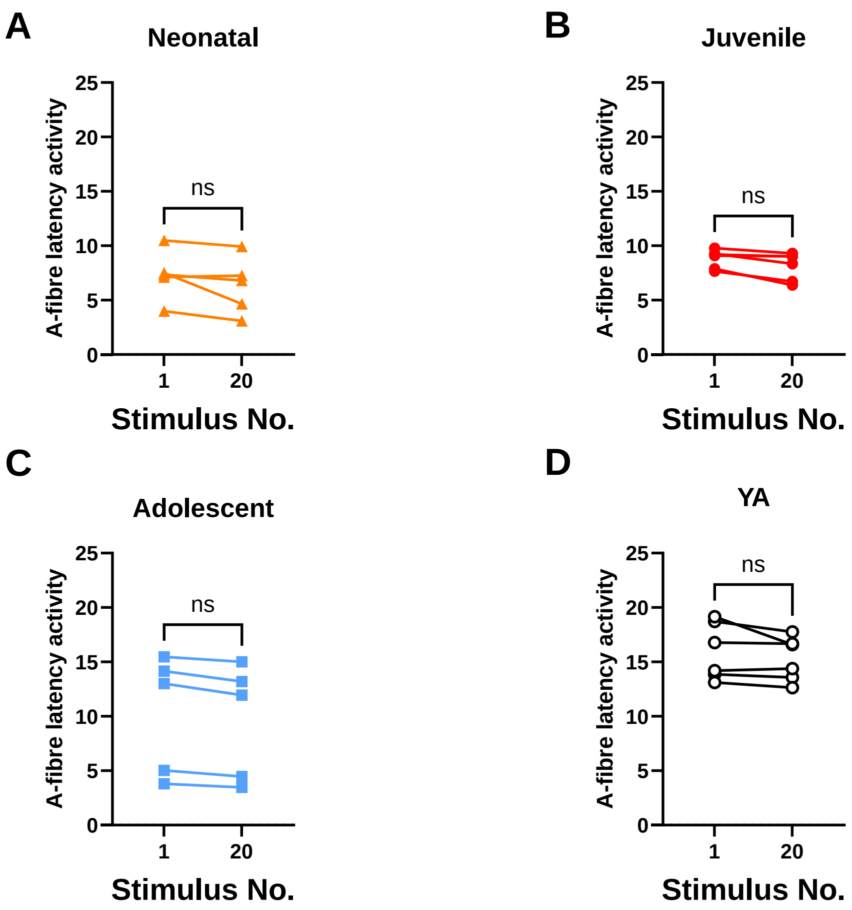

Supplementary Figure 3: Lack of sensitization of A fibre latency responses following repeated high frequency stimulation of the hindpaw in all age groups. Responses did not vary significantly within longer trains of repeated high frequency, high amplitude stimulation (5mA, 20x 2ms, 0.5Hz) in neonatal (**A**), juvenile (**B**), adolescent (**C**), or young adult (**D**) animals. Data represent summed threshold crossings in the A latency period averaged across the whole array. Add 1mA 1st vs 5th for all age groups from electrical data
